# Giant Transposons in Eukaryotes: Is Bigger Better?

**DOI:** 10.1093/gbe/evz041

**Published:** 2019-02-23

**Authors:** Irina R Arkhipova, Irina A Yushenova

**Affiliations:** Josephine Bay Paul Center for Comparative Molecular Biology and Evolution, Marine Biological Laboratory, Woods Hole, Massachusetts

**Keywords:** transposable elements, transposition, mobile DNA, reverse transcriptase, transposase

## Abstract

Transposable elements (TEs) are ubiquitous in both prokaryotes and eukaryotes, and the dynamic character of their interaction with host genomes brings about numerous evolutionary innovations and shapes genome structure and function in a multitude of ways. In traditional classification systems, TEs are often being depicted in simplistic ways, based primarily on the key enzymes required for transposition, such as transposases/recombinases and reverse transcriptases. Recent progress in whole-genome sequencing and long-read assembly, combined with expansion of the familiar range of model organisms, resulted in identification of unprecedentedly long transposable units spanning dozens or even hundreds of kilobases, initially in prokaryotic and more recently in eukaryotic systems. Here, we focus on such oversized eukaryotic TEs, including retrotransposons and DNA transposons, outline their complex and often combinatorial nature and closely intertwined relationship with viruses, and discuss their potential for participating in transfer of long stretches of DNA in eukaryotes.

## Introduction

The distinguishing characteristic of transposable elements (TEs), or mobile genetic elements (MGEs), is the ability to change their chromosomal location, not only within, but also between genomes, as well as between species or even higher-order taxa. The terms MGEs and TEs are fully interchangeable, but for historical reasons use of MGE was mainly adopted by the prokaryotic community and TE—by the eukaryotic community; we maintain this subdivision here for convenience only. These discrete segments of DNA typically code for mobility-related enzymatic functions, which come in several different flavors, but for the most part enable breakage and joining of chromosomal DNA (Craig et al. 2015; Arkhipova 2017). Nonautonomous TEs, which carry the *cis*-acting sequences required for transposition, can relocate within and between genomes only if the necessary enzymatic functions are provided in *trans* by autonomous elements encoding the required transposition machinery.

In addition to the “selfish” function of multiplying themselves, and thereby ensuring their own survival, TE-encoded functions may also support mobilization of unrelated fragments of genomic DNA, if these fragments are appropriately positioned between *cis*-acting elements or otherwise placed within genomic DNA segments that will be subject to relocation. Intergenomic mobilization of genetic material results in a nonvertical mode of their inheritance, that is, horizontal gene transfer (HGT).

In bacteria and archaea, much attention has been paid to MGEs as potential HGT vehicles. It is well known that HGT plays a major role in evolution of prokaryotic genomes, with a good three-quarters of bacterial genes estimated to have undergone at least one HGT event at some point in evolution (Kloesges et al. 2011; Koonin 2015; Soucy et al. 2015). Initially, HGT events were observed through the emergence of specific phenotypes conferred by horizontally transferred determinants, for example, antibiotic resistance, virulence, or heavy metal tolerance. These determinants were mostly carried on mobile vehicles, such as plasmids or phages, which can accommodate substantial amounts of extra genetic material. As the emphasis in discovery shifted from cultivation-based experimental approaches to whole-genome shotgun sequencing, so did the identification of such determinants. It now starts with bioinformatic scanning and comparison of entire bacterial genomes to identify all possible candidate loci with the potential to confer specific phenotypes.

In eukaryotes, TEs occupy a significantly larger fraction of genomic DNA than in prokaryotes, and could make up to 70% of the genome in vertebrates and over 80% in plants (Sotero-Caio et al. 2017; Wicker et al. 2017; Rodriguez and Arkhipova 2018), often to the point when they turn into an assembler’s nightmare (Rogers et al. 2018). Their intrinsic capacity to relocate autonomously not only within but also between genomes frequently results in their horizontal expansion throughout populations, species, genera, and higher-order taxonomic groups. Such lateral exchange occurs on a considerable scale and is often referred to as horizontal transposon transfer (Schaack et al. 2010; Peccoud et al. 2017; Wallau et al. 2018). Eukaryotic genomes are known to undergo HGT (Andersson 2005; Keeling and Palmer 2008; Boto 2014), albeit much less frequently than prokaryotic ones, due to the existence of a well-protected germ line in metazoans. Thus, the role of HGT in eukaryotic evolution has often been dismissed as insignificant (Ku et al. 2015; Martin 2017). Even more mysterious are the mechanisms possibly mediating interkingdom and/or interdomain gene transfers. Although viruses have been invoked as HGT vectors in cross-species transfers (Piskurek and Okada 2007; Gilbert and Cordaux 2017), and host–parasite and/or endosymbiotic interactions have been argued to facilitate HGT across phyla (Gilbert et al. 2010; Sieber et al. 2017), the capacity of the eukaryotic mobilome, that is, the sum of all mobile elements, to drive lateral transmission of genetic material remains largely understudied, due in part to our incomplete understanding of TE diversity and their mobilization potential.

The technological advances resulting in generation of increasingly long stretches of eukaryotic DNA have recently improved our potential to identify large units of mobility, the size of which may significantly exceed the previously known size limits for eukaryotic TEs. By analogy to the large mobilizable units operating in the bacterial world, it may be argued that lateral gene transfer in eukaryotes could be associated with mobile entities capable of accommodating cargo loads of higher-than-expected capacity. Here, we review the main types of oversized eukaryotic TEs which were identified in recent years through comparative analysis of large chromosomal DNA segments. Further, we attempt to evaluate their potential for intra- and intergenomic mobility and the associated movement of cargo genes and/or gene blocks. To facilitate comparisons for researchers primarily studying eukaryotic genomes, we begin with a brief overview of large prokaryotic MGEs, which are typically associated with HGT.

## Large Mobile Elements in Bacteria

Each of the three pillars of lateral gene exchange in bacteria—conjugation, transduction, and transformation—has, in one way or the other, been connected to various components of the bacterial mobilome. Many excellent reviews have been devoted to bacterial HGT, for example, a recent comprehensive collection in a special issue of *Current Opinion in Microbiology* (Lang, Beatty, et al. 2017).

The earliest described cases of antibiotic resistance were associated with plasmids, many of which harbored insertion sequences conferring mobility to a resistance determinant contained in between, forming a larger composite transposon (Tn) framed by two insertion sequence elements. Although a lot of small plasmids can replicate autonomously but do not encode their own conjugation systems, many of them, along with their cargo, can in fact be mobilized by larger conjugative plasmids carrying the genes for mating-pair formation, including Type IV secretion system responsible for transfer of single-stranded DNA (ssDNA) between cells, as well as conjugative relaxases recognizing the *cis*-acting origin of transfer (*oriT*) (Ramsay and Firth 2017). Closely related to conjugative plasmids are the integrative and conjugative elements (ICEs), or conjugative transposons, which in addition to the conjugative apparatus have acquired the capacity to integrate into the chromosome, conferred by an element-encoded recombinase. Although most ICE-encoded recombinases belong to the tyrosine recombinase family, some belong to serine recombinases, with both families recognizing the *attP/attB* sites in the ICE/target, respectively, or to DDE transposases, which recognize terminal inverted repeats (TIRs) at the MGE termini (Wozniak and Waldor 2010; Johnson and Grossman 2015; Burrus 2017). The size of ICEs can vary from 20 to >500 kb; some well-studied examples include *Tn916* (18 kb), *Tn5397* (21 kb), and *CTnDOT* (65 kb) (Johnson and Grossman 2015; Wood and Gardner 2015). Further size increases are largely enabled by the modular ICE nature, which allows variable representation of conjugative, integration/excision, regulatory, and cargo gene modules in these mosaic elements. Some of the previously recognized genomic islands (GIs), large segments of DNA displaying signs of prior mobility, are mobilizable as nonautonomous ICEs. Most intriguingly, huge ICEs harboring GIs (>500 kb total) were shown to exist as three separate chromosomal regions, assembling into a single circle for conjugative transfer by sequential action of three recombinases aided by directionality factors (Haskett et al. 2016, 2018).

Other types of GIs can be mobilized by transduction, rather than conjugation, and depend on the associated helper phages for intergenomic transfer. Phage-related MGEs are represented by phage-inducible chromosomal islands (PICIs), including staphylococcal pathogenicity islands (Penades and Christie 2015; Novick and Ram 2016), as well as by gene transfer agents (Lang et al. 2012; Lang, Westbye, et al. 2017). In contrast to the genuine phage-mediated generalized or specialized transduction (Touchon et al. 2017; Toussaint and Rice 2017), PICIs hijack the phage-encoded functions and prevent the propagation of the helper phage by interfering with proper capsid formation (Novick and Ram 2017). The resulting capsids are much smaller and unable to accommodate the entire phage genomes, packaging only 15–30 kb of DNA carrying the cargo and the functions needed for integration and helper phage suppression, instead of 45–100 kb typical for phages. Interference with late phage gene transcription is another PICI strategy to suppress the helper phage. Gene transfer agents differ from other GIs in that they do not encode any phage-related functions but rely on the “domesticated” chromosomal set of phage-related genes to package and transfer relatively short (4–14 kb) random fragments of genomic DNA (Lang et al. 2012; Lang, Westbye, et al. 2017). Interestingly, delivery of transduced DNA cargo in this case is achieved by the natural transformation system (called Com) encoded in the recipient (Lang, Westbye, et al. 2017).

Transformation, ensured by the natural competence system, is usually thought to be reserved for uptake of smaller DNA molecules from the environment, such as small plasmids and fragments of chromosomes. However, transfer of much longer fragments (7–45 kb) has been recorded (Blokesch 2017). It has been argued that biases during transformation to replace insertion-bearing loci with shorter empty sites rather than the other way around have evolved in bacteria as an adaptation to intragenomic conflict with MGEs, which can in turn disrupt multiple competence genes (Croucher et al. 2016).

Finally, a special type of MGEs called integrons, involved in capture and transmission of multiple determinants of resistance to antibiotics or other selective agents, displays a structure defined by a tyrosine recombinase and the adjacent *att* site, which is capable of incorporating, accumulating and expressing gene cassettes from its own promoter (Gillings 2017). Although integrons do not constitute autonomous mobile units, they can spread between genomes by using conjugative plasmids or transposons. Remarkably, although a single cassette (<1 kb) would typically carry only one open reading frame (ORF), hundreds of cassettes can be sequentially arranged into chromosomal superintegrons, which may exceed 100 kb in length and occupy up to 10% of a given bacterial genome (Rowe-Magnus et al. 1999).

Overall, the ability of prokaryotes to mobilize and exchange long stretches of DNA is well-established and has a significant impact on genome evolution. It is also worth noting that most genetic exchanges take place at the DNA level via DNA breakage and joining, as necessitated by the predominantly circular nature of bacterial chromosomes.

## Known TE Types in Eukaryotes: Size Limitations and Assembly Difficulties

The cargo carried by large bacterial MGEs includes a multitude of nonessential genes providing adaptations to specific niche environments: factors conferring resistance to antibiotics, heavy metals, aromatic compounds; virulence factors; genes involved in pathogen–host interaction; etc. Except for certain challenges characteristically faced by bacteria, such as nitrogen fixation or biofilm formation, many of the free-living microscopic eukaryotes are facing quite similar challenges in their environments. It may therefore be asked whether some of the eukaryotic genomes may harbor TEs with analogous properties that could accommodate and transmit similar determinants.

In fungi (*Fusarium* spp.), entire lineage-specific “pathogenicity chromosomes” consisting exclusively of pathogenicity-related genes and TEs can transfer between strains and ensure virulence against specific plant hosts (Ma et al. 2010). Evidently, the ability to function and segregate as a chromosome would be conferred in *cis* by essential sequence elements such as centromeres and telomeres, whereas the exact transfer mechanisms remain to be determined. However, the majority of known eukaryotic TE types, including those that captured host genes, fall into much more modest size ranges, because introduction of large blocks of nonhomology into diploid eukaryotes potentially introduces problems during meiosis, especially when present in nonhomologous locations on chromosomes. Nevertheless, by comparing the mobilomes of bacteria and eukaryotes, and focusing on those TEs which can tolerate substantial cargo loads, we may be able to observe preferential capture and spread of extra genetic material by certain types of TEs in comparison with the others.

Continuing progress in whole-genome sequencing technologies in recent years led to realization that the size limitations previously placed on mobilizable DNA units in eukaryotes may have been due in part to our inability to assemble large contiguous stretches of repetitive DNA in complex genomes. The limited contiguity provided by Illumina mate-pair libraries, which typically do not exceed 20 kb, has previously kept most of the oversized TEs under the radar. Nowadays, with the availability of third-generation sequencers such as PacBio SMRT sequencers (Pacific Biosciences) and MinION nanopore devices (Oxford Nanopore), contiguous long reads tens of kilobases in length can reach across oversized TEs, helping to uncover a hitherto unappreciated structural and coding diversity of giant mobilizable units. Thus, a reassessment of our current understanding of TE ability to increase in size would be timely.

**Fig. 1.**
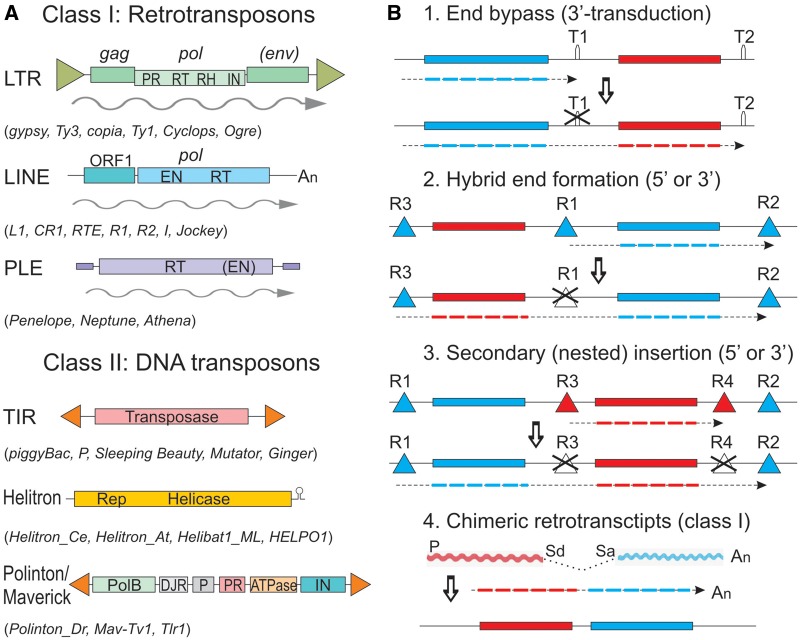
—Structure of eukaryotic TEs and models for gene capture. (*A*) A traditional simplistic representation of TE structural organization. PR, protease; RT, reverse transcriptase; RH, RNase H; IN, integrase; EN, endonuclease; An, poly(A); PolB, family B polymerase; DJR, double jelly-roll capsid protein; P, penton capsid protein. RNA intermediate is denoted by a wavy line. Examples are listed in parentheses; adapted from Wicker et al. (2007), Sotero-Caio et al. (2017), and Rodriguez and Arkhipova (2018). (*B*) Some of the proposed models for gene capture by eukaryotic TEs. TE-associated terminal repeats (R) with *cis*-acting elements are denoted by triangles, and their orientation (either direct or inverted) can be assigned depending on the nature of the TE. T, termination signals for transcription or replication; P, promoter; Sd, splice donor; Sa, splice acceptor. Captured genes are in red. Dashed lines denote transposition intermediates, either in DNA or RNA form; adapted from Kapitonov and Jurka (2007) and Thomas and Pritham (2015).

Many known TEs are capable of capturing genes and/or gene fragments, and this ability is not restricted to a specific mode of replication characterizing retrotransposons (class I) or DNA TEs (class II) (Fig. 1*A* and *B*). Beginning with the discovery of *onc* gene capture by RNA tumor viruses (i.e., retroviruses) in the 1970s, host gene transduction by endogenous retroviruses and the structurally similar long terminal repeat (LTR) retrotransposons has occasionally been reported in diverse animals and plants (Stehelin et al. 1976; Du et al. 2010; Steinbauerová et al. 2011; Jiang and Ramachandran 2013; Chong et al. 2014; Rodriguez et al. 2017). Other, nonviral retrotransposons are not immune to transduction either: for example, host DNA can be transduced by L1 retrotransposons of the non-LTR subclass, or by *Penelope*-like elements (PLEs) (Fig. 1*A*) (Goodier et al. 2000; Pickeral et al. 2000; Arkhipova et al. 2013). However, the fragments 3′-transduced by L1 rarely exceed 1 kb in humans or 3 kb in mice, as transduction is usually limited by the distance to the next available poly(A) signal in the adjacent DNA (Fig. 1*B*). For LTR retrotransposons and PLEs, the capacity to transfer material between terminal repeats appears somewhat higher. A 4-kb nonribosomal peptide synthetase module was transposed using PLE terminal repeats as *cis*-acting elements, generating a 8-bp target-site duplication upon insertion (Arkhipova et al. 2013). LTR retrotransposons can capture occasional ORFs downstream of the *pol* gene: in lieu of *env*, downstream of *env*, or between *pol* and *env* (Du et al. 2010; Steinbauerová et al. 2011; Chong et al. 2014; Rodriguez et al. 2017) (Fig. 1*A* and *B*). Additionally, DNA fragments, not necessarily coding, can become trapped between two LTRs, replacing much of the original TE coding sequence, and then relocated nonautonomously in *trans*, forming the so-called large retrotransposon derivatives (Kalendar et al. 2004). In the above cases, the total length of the genetic material contained between two LTRs, be it a combination of retrotransposon and host ORFs or just the host DNA transduced nonautonomously, rarely exceeds 10 kb.

Among DNA TEs (class II), certain superfamilies appear to be particularly prone to gene fragment capture. Pack-MULEs, *Mutator*-like DNA TEs found in plants, are “packed” with gene fragments enclosed between TIRs, and are mobilized by a cut-and-paste mechanism using a *MuDR* DDE transposase provided in *trans* (Jiang et al. 2004, 2011). If compared with bacterial MGEs, these elements would most closely resemble the above-mentioned large ICEs called Tn*GBS*, containing a *Mutator*-like transposase which recognizes and employs TIRs for mobilization (Guérillot et al. 2014). In the red alga *Chondrus crispus*, significantly enlarged (16–20 kb) En/Spm (also called CACTA) TE families harboring 4–5 ORFs instead of the usual 1–2 ORFs were reported (Kojima et al. 2013). *Helitrons*, which transpose by rolling-circle replication, can also capture multiple gene fragments by a variety of proposed mechanisms (Fig. 1*B*) (Morgante et al. 2005; Kapitonov and Jurka 2007; Thomas and Pritham 2015); most of these models are applicable to both DNA- and RNA-based events. Nevertheless, the length of the transposing DNA segment that can be naturally accommodated by DNA TEs in eukaryotes might be subject to certain limitations. In fact, none of the Pack-MULEs reported by Jiang et al. (2004) exceeded the full-length 7-kb element, most being in the range of 1–2 kb. This may be related to the fact that the efficacy of DNA transposition drops significantly over 6 kb of cargo size, apparently interfering with transfer of large gene blocks in nature, although DNA cargoes 100–200 kb in length were mobilized in artificial *Sleeping Beauty*-, *piggyBac*-, or *P-element*-based transgenesis constructs (Ring et al. 2000; Li et al. 2013; Narayanavari et al. 2017). In sum, although technological advances are helping us to uncover increasingly large units of mobility and to manipulate longer inserts, natural barriers to expansion may be acting to keep eukaryotic TEs as lean as possible.

## Large Eukaryotic TEs: Raising the Limits, Breaking the Barriers?

As mentioned above, long stretches of DNA in nonhomologous locations increase the probability of ectopic recombination events, which are strongly selected against. The efficiency of DNA transposition also drops with increasing distance between TIRs, disfavoring larger inserts unless they are selected for. Also, both class I and class II TEs serve as targets for the RNA-mediated silencing machinery, which restricts the expression of active TEs. In retrotransposons, additional factors limiting the total length may include 1) issues with producing a large full-length transcript, 2) the ability of reverse transcriptase (RT) to perform processive cDNA synthesis, and 3) by the packaging capacity of ribonucleoprotein particles carrying the long transcript. These limits have traditionally revolved around 10 kb, and anything longer than that was labeled as giant despite the canonical ORF composition, for example, LTR retrotransposons *Cyclops* (12 kb) or *Ogre* (25 kb) (Chavanne et al. 1998; Macas and Neumann 2007). In this review, we will consider TEs over 20 kb long and apply the limit to the internal coding region only, to avoid counting in duplicate any long noncoding terminal repeats, while focusing on the excess coding capacities of large TEs. In the following subsections, we will concentrate on several TE families, first on retrotransposons and then on DNA transposons, to understand how these TEs managed to circumvent any potential size limits and whether inclusion of extra DNA might translate into rare events that could eventually result in transmission of long DNA blocks in a nonvertical fashion.

### Giant Gypsy-like LTR Retrotransposons in Planarians

**Fig. 2.**
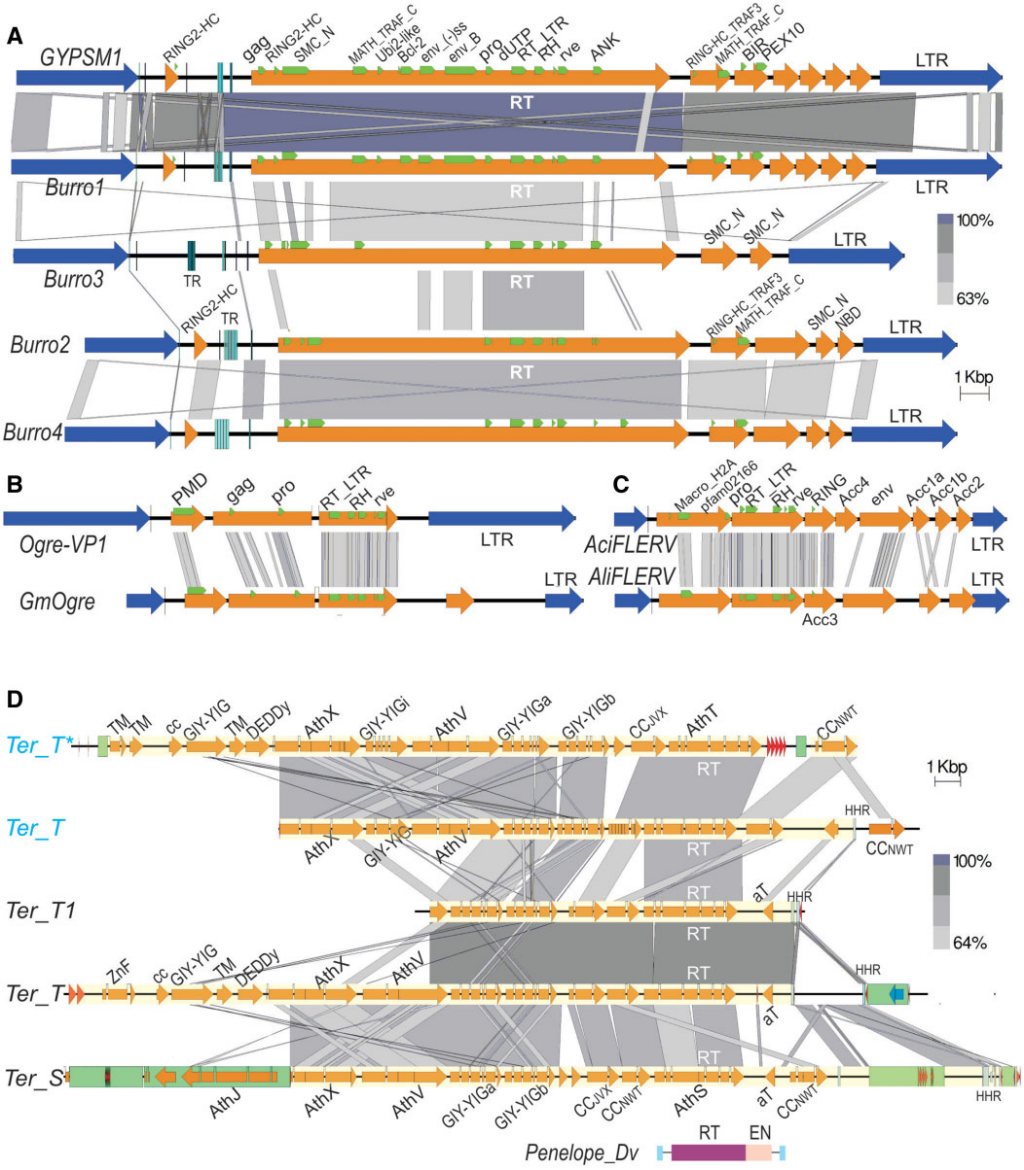
—Structure of giant retrotransposons in invertebrates. (*A*) Gypsy-like *Burro* LTR retrotransposons in the planarian *Schmidtea mediterranea* (Jurka 2007; Grohme et al. 2018). LTRs, blue arrows; ORFs, yellow arrows; conserved motifs mentioned in the text, green frames; vertical bars, tandem repeats (TR). (*B*) The longest *Ogre* LTR retrotransposons from soybean *Glycine max* (*GM-Ogre*) and vetch *Vicia pannonica* (*Ogre-VP1*) (Macas and Neumann 2007; Laten et al. 2008) are drawn to the same scale for comparison. (*C*) Foamy-like endogenous retroviruses from the midas cichlid *Amphilophus citrinellus* (*AciFLERV*) and from annual killifish *Austrofundulus limnaeus* (*AliFLERV*) (Aiewsakun and Katzourakis 2017); same scale. (*D*) Giant *Terminon* retroelements in the bdelloid rotifer *Adineta vaga* (Arkhipova et al. 2017); two homologous members of the same *Ter_S/T* family from the natural *A. vaga* isolate are labeled in blue. Blue vertical lines, hammerhead ribozyme motifs (HHR); green boxes, secondary TE insertions; *defective copy. For comparison, domain structure of the canonical *Penelope* retrotransposon from *Drosophila virilis* (Evgen'ev and Arkhipova 2005) is drawn to the same scale. All panels were drawn with Easyfig 2.2.2 (Sullivan et al. 2011). Regions of sequence homology are connected with gray-shaded boxes; the intensity of shading corresponds to percent BlastN (*A, D*) or TBlastN (*B, C*) identity, as indicated. Scale bar, 1 kb.

In the planarian *Schmidtea mediterranea*, the size records were recently broken by giant gypsy-like LTR retrotransposons, present in thousands of copies (Grohme et al. 2018) (Fig. 2*A*). Their internal region measures up to 25 kb, and the total exceeds 30 kb with LTRs included, prompting the authors to name these elements *Burro* (big unknown repeat rivalling *ogre*) and dethroning the 16–25 kb *Ogre* retrotransposons of dicot plants (Macas and Neumann 2007). However, the claim to gigantism in *Ogre* was mostly attributable to the extraordinary LTR length (1.9–6.5 kb), which is comparable with the length of the 11–13 kb internal region carrying 1–2 extra ORFs of unknown function (Fig. 2*B*). It should be noted that *Burro1* was previously described under the name *GYPSM1* (Fig. 2*A*; 97% identity to *Burro1*) by Jerzy Jurka, who noted that it “carries several very interesting protein motifs and might have played a role as an evolutionary tinkerer” (Jurka 2007). He pointed out that in addition to the protease (PR)–RT–RNase H (RH)–integrase (IN) (Fig. 1*A*), it harbors such unusual motifs as AIR1 (arginine methyltransferase-interacting protein with RING Zn-finger), Smc (structural maintenance of chromosomes), MATH_TRAF_C (meprin and TRAF-C homology, often involved in protein processing and ubiquitination [Zapata et al. 2007]), BIR (baculoviral inhibition of apoptosis protein repeat domain) and ankyrin repeats (Fig. 2*A*). These and a BCL2-like (apoptosis regulator protein) motif were also noted in *Burro-2, -3*, and *-4* elements (Grohme et al. 2018). However, the above domains occupy only one-half of the enormous 4,873-aa ORF1 polyprotein. We therefore undertook further domain annotation through HHpred comparisons (Zimmermann et al. 2018) with an intact *Burro*-like element reconstructed from another planarian, *Dugesia tigrina* (not shown). Its 5,140-aa ORF1 lacks the MATH_TRAF_C domain, which is encoded by an upstream accessory ORF; instead, it reveals homology to midasin (MDN1), an AAA ATPase involved in ribosome maturation. Notably, the N-terminus in both species can be unambiguously classified as a typical Gag, with the retrotrans_gag motif followed by three (*S. mediterranea*) or two (*D. tigrina*) CCHC Zn-knuckles. Other motifs of interest are Ubi2/SUMO-like (PF11976) between MATH_TRAF_C and BCL-2, and dUTPase-like (PF00692) between PR and RT (Fig. 2*A*), also seen in this position in LTR retrotransposons from fungi and rotifers and in various positions in many retroviruses (Riccioni et al. 2008; Hizi and Herzig 2015; Rodriguez et al. 2017). Most interestingly, the region between BCL2 and PR carries homology to *gypsy* Env (96.7% probability), which in turn shares homology with fusion glycoproteins of baculoviruses, retroviruses, and paramyxoviruses. The *env* region (Fig. 2*A*) yields multiple hits to fusion glycoproteins from arthropod and fish mononegaviruses. This is the first case of *env* in-frame localization between *gag* and *pol*, whereas in other gypsy-like TEs it is expressed as a separate ORF.

The finding of *env* homology lends support to a hypothesis that *Burro*-like retrotransposons represent integrated proviruses, which have been actively shaping planarian genomes in the family Dugesiidae. The third sequenced species in the family, *Dugesia japonica*, contains decaying fragments of *Burro* elements with stop codons and frameshifts. Their highly unusual ORF structure offers a unique example of integrating multiple domains into a humongous polyprotein encoding diverse enzymatic, structural and regulatory functions, and harboring accessory ORFs which may alternatively exist as subdomains within the polyprotein. Accessory ORFs led to significant size increases (up to 17 kb) in foamy-like fish endogenous retroviruses (Aiewsakun and Katzourakis 2017), which otherwise display a more conventional retrovirus-like structure (Fig. 2*C*). The prominence of extra domains associated with regulation of apoptosis and ubiquitination indicates that *Burro* might indeed be prospering as a tinkerer meddling with the corresponding host systems—a possibility worthy of experimental confirmation, notwithstanding the difficulties of studying such a complex TE in an emerging model organism.

### Terminons: Giant Retroelements at Rotifer Telomeres

Retroelements of the PLE subclass, belonging to a distinct RT clade which shares common ancestry with telomerase RTs (Arkhipova et al. 2003), have until recently been regarded as quite compact. Either with a single ORF including the RT and the GIY-YIG endonuclease (EN) domains, or with two ORFs lacking the EN domain, they typically measured 4–6 kb in length (Arkhipova 2006; Gladyshev and Arkhipova 2007; Lin et al. 2016). This view has drastically changed with the realization that, in the context of whole-genome sequence, some of the ∼6-kb RT-encoding mobile units in cloned telomeres of bdelloid rotifers (Gladyshev and Arkhipova 2007) represented only the 3′-terminal fragments of giant retroelements up to 40 kb in length (Arkhipova et al. 2017) (Fig. 2*D*). These EN-deficient elements, in addition to RT, can accommodate a variety of accessory functions, such as DEDDy 3′-exonucleases, GDSL esterases/lipases, GIY-YIG-like ENs, rolling-circle replication initiator (Rep) proteins, and putatively structural ORFs with coiled-coil motifs and transmembrane domains. Most of the copies are 5′-truncated by short stretches of telomeric repeats and can form long head-to-tail tandem or interspersed arrays, with host genes often captured between neighboring TEs.

Most intriguingly, the 3′-ends of these large elements, called *Terminons*, have apparently developed the ability to attach to the exposed G-rich telomeric repeats at the chromosome ends by incorporating a short stretch of reverse-complement telomeric repeats immediately downstream of the characteristic 3-terminal fold, the hammerhead ribozyme motif (HHR). The HHR motif can also be found within terminal repeats of nontelomeric PLEs (Cervera and De la Pena 2014), but only the telomere-associated PLEs provide the unique 3′-terminal structure in which the ribozyme fold exposes the adjacent (ACACCC)_n_ or (TCACCC)_n_ stretches to facilitate base-pairing with the corresponding G-rich overhangs at the chromosome termini (Arkhipova et al. 2017). If we consider retroelement composition from the viewpoint of combining the principal functions of polymerization, integration, and host–TE interaction (Arkhipova 2017), the cleavage function of the HHR motif may be regarded as auxiliary for preprocessing of RNA templates, and the EN function would be dispensable if no cleavage of internal chromosome regions is required. Subsequent build-up of long subterminal chains may further lead to break-induced replication, chromosome fission/fusion, and formation of internalized GIs.

If accessory ORFs within the units participate in the retrotransposition cycle through facilitating intra-or intercellular trafficking, helping to subvert host defenses, or benefitting the TE in some other way, one may entertain several nonconventional aspects of their utilization, in light of the 3D TE composition scheme described by Arkhipova (2017). If the polymerization function is assigned to the RT-encoding ORF and the integration function to the 3′-terminal HHR-telomeric repeat structure, the possibilities for horizontal exchange may be augmented by the GDSL esterase/lipase conferring the ability to penetrate through cell membranes, which may also be applicable to LTR retrotransposons (Rodriguez et al. 2017); DEDDy-like exonucleases could assist in 3'-end processing of structured RNAs or ssDNAs; stand-alone GIY-YIG ENs could facilitate the initial integration of master copies; and association with Rep proteins may point at the existence of circular intermediates. Furthermore, detection of HHRs in circular RNAs of nonautonomous plant LTR elements called retrozymes strengthens the links between retroelements and HHR-containing viroids—small subviral RNAs in plants propagating in a circular form (Cervera et al. 2016).

### Helitrons: DNA Transposons Prone to ORF Capture

**Fig. 3.**
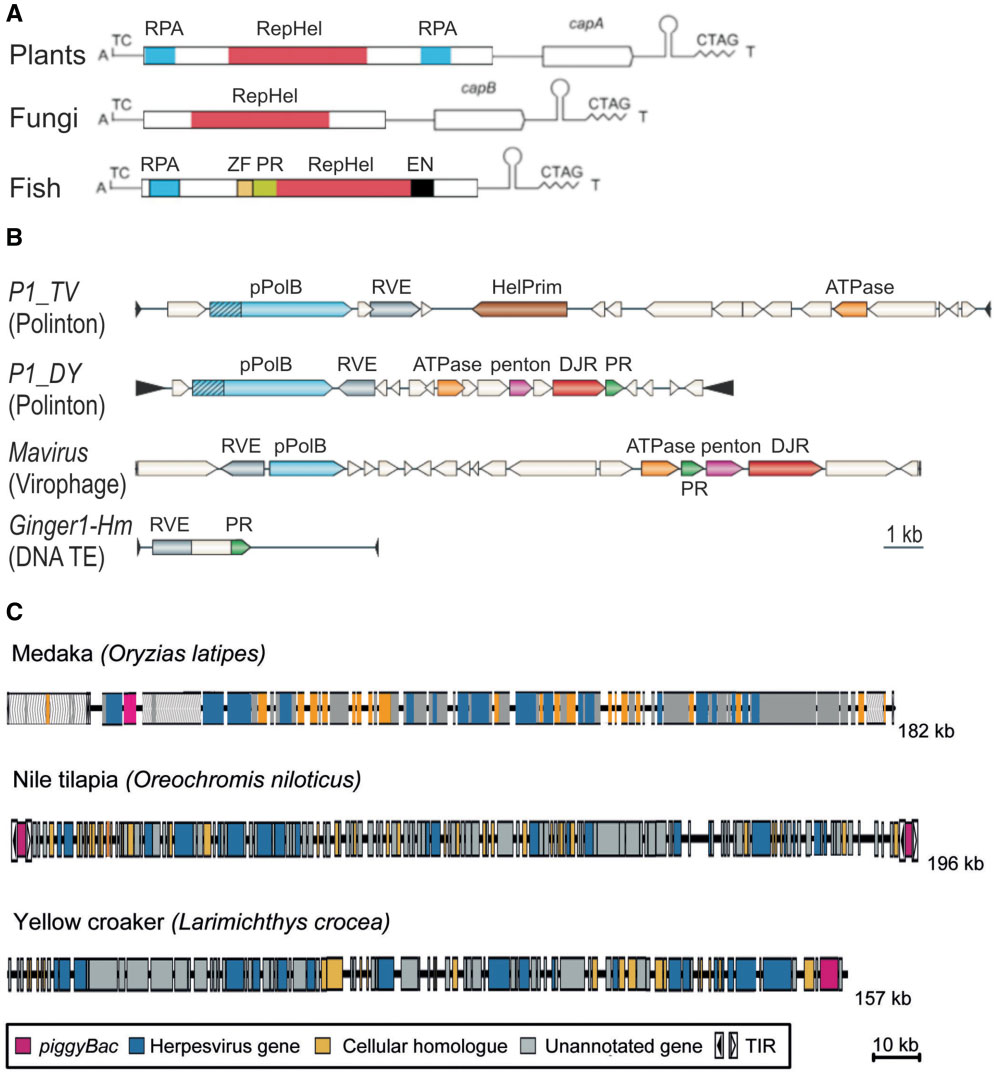
—Structural organization of large DNA transposons from the *Helitron*, *Polinton/Maverick*, and *Teratorn* superfamilies. (*A*) Examples of *Helitrons* from plants, fungi, and fish; adapted from Kapitonov and Jurka (2007) and Castanera et al. (2014). PFAM domains are described in the text; capA-B, captured gene fragments; terminal nucleotides and hairpin structures are indicated. (*B*) Comparison of *Polintons* from *Trichomonas vaginalis* (*P1_TV*) and *Drosophila yakuba* (*P1_DY*) with the *Mavirus* virophage from *Cafeteria roenbergensis* and *Ginger1-4* DNA TE from *Hydra magnipapillata*; adapted from Krupovic and Koonin (2015). Colored ORFs are marked as described in the text; TIRs are denoted by triangles. Scale bar, 1 kb. (*C*) *Teratorn*-like elements from medaka (*Oryzias latipes*), Nile tilapia (*Oreochromis niloticus*), and yellow croaker (*Larimichthys crocea*); adapted from Inoue et al. (2017). Predicted ORFs (exons) are depicted by colored rectangles according to the categories indicated in the box. Scale bar, 10 kb.

Moving from retrotransposons to DNA transposons, we first consider *Helitrons*, which transpose by the rolling-circle replication mechanism. What places them on the larger side of the eukaryotic TE size range is the length of the main ORFs involved in transposition: The RepHel protein with rolling-circle replication initiator (Rep) and helicase (Hel) domains measures over 1,500 aa in length, and can exceed 2,000 aa if additional domains are present, for example, ssDNA-binding protein RPA in plant, cnidarian and fish *Helitrons*, or OTU-like cysteine PR and AP-EN in animal and certain fungal *Helentrons* (Fig. 3*A*) (Kapitonov and Jurka 2007; Thomas and Pritham 2015). Transposition occurs via a circular double-stranded DNA intermediate, but only the (+) strand is transposed, as in circular ssDNA viruses (Grabundzija et al. 2018). *Helitrons* are known to capture and transduce host gene fragments, especially in plants (Morgante et al. 2005), but also in animals and fungi. In fungi, their cumulative length could increase from the typical 6–7 to 14 kb, and in maize up to 39 kb, capturing up to nine gene fragments (Du et al. 2009; Castanera et al. 2014). The prevailing mechanistic explanation for acquisition of host gene fragments by *Helitrons* is the bypass of a hairpin structure serving as a terminator of rolling-circle replication, and the use of the next available downstream signal from the host or a downstream *Helitron* (Kapitonov and Jurka 2007; Grabundzija et al. 2016), much like it happens during 3′-transduction by L1 retroelements (Szak et al. 2003) (Fig. 1*B*). Furthermore, *Helitrons* were found in polydnaviruses, sometimes comprising entire viral segments (Thomas and Pritham 2015; Heringer et al. 2017). However, as in Pack-MULEs, acquisition of full-length host genes rather than gene fragments by *Helitrons* is exceptionally rare, limiting their potential as agents for gene dissemination.

### Polintons/Mavericks: DDE INs and Viral Connections

These self-replicating DNA TEs, found in diverse eukaryotes from protists to vertebrates, encode a protein-primed family B DNA polymerase (PolB) for replication, and a retroviral-like DDE IN for integration into host DNA (Fig. 3*B*) (Kapitonov and Jurka 2006; Pritham et al. 2007). They surpass *Helitrons* in length and coding potential, and are rightfully classified as giant, typically measuring 15–25 kb in length, with representatives reaching 40 kb. Such extraordinary length is achieved through orientation-independent accumulation of 5–10 ORFs, of which the most conserved are the adenovirus-like cysteine PR and the packaging ATPase. Their similarity to adenoviruses, bacteriophages, and linear eukaryotic plasmids suggested an evolutionary connection between these disparate groups. Indeed, a *Mavirus* virophage parasitizing on a giant virus of a marine flagellate *Cafeteria roenbergensis* is closely related to *Mavericks/Polintons* (Fischer and Suttle 2011), and many *Polintons* encode major and minor jelly-roll capsid-like proteins, earning them the name “Polintoviruses” (Krupovic et al. 2014). Network analysis placed *Polintons* at the core of the evolutionary transition between bacteriophages and eukaryotic selfish genetic elements such as linear plasmids and double-stranded DNA viruses (adenoviruses, virophages, and giant megabase-sized nucleocytoplasmic DNA viruses of the order Megavirales) (Krupovic and Koonin 2015). Such transition was presumably made possible by acquisition of PR and DDE IN by an ancestral phage, with *Ginger*-like DNA TEs cited as a possible source of these two activities (Bao et al. 2010; Krupovic and Koonin 2015). Subsequent loss/gain of components may have led to further diversification into plasmids or viruses. The ability to colonize eukaryotic genomes, conferred by the IN and the corresponding TIRs, allowed them to spread throughout chromosomes, sometimes occupying up to 30% of the genome, as in the protist *Trichomonas vaginalis* (Pritham et al. 2007).

It has been argued that protein-primed PolB could impose size limits on the length of the corresponding replicons (hypothesized to cap at ∼45 kb, e.g., in adenoviruses), and that its replacement with a DNA/RNA-primed PolB and coupling with a helicase–primase opened the route to large-scale genome expansion in Megavirales (Krupovic and Koonin 2015). On this view, combining a nucleic acid-primed polymerase with a primase could potentially give rise to novel TEs of unprecedented sizes upon acquisition of a suitable IN/recombinase.

### Teratorn: A Herpesvirus piggyBac(k)Ing for Integration

A DDE IN from a different superfamily, related to those found in *piggyBac* DNA TEs, was instrumental in converting a fish herpesvirus into a novel TE. *Teratorn* is an active mobile element arising from fusion between *piggyBac*-like DNA TE and a herpesvirus from the family Alloherpesviridae (Inoue et al. 2017). Discovered in a small teleost fish medaka (*Oryzias latipes*), *Teratorn* was soon found in seven other teleosts (yellow croaker, Nile tilapia, ocean sunfish, turquoise killifish, annual killifish, Atlantic salmon, Coho salmon, and Asian swamp eel), although relative location of herpesvirus genes and the *piggyBac*-like transposase suggests that its acquisition occurred on multiple occasions (Inoue et al. 2017, 2018).

*Teratorn* is a giant (41–182 kb long) mobile element equipped with an active transposase and 18-bp TIRs. Interestingly, *Teratorn* possesses additional TIRs at the boundary of a pair of long inverted repeats and a unique region, that is, “internal TIRs.” The external TIRs are less frequently used for integration than internal TIRs. The two subtypes of *Teratorn* identified in the medaka genome are similar in structure, except for an 80-kb inversion in the middle. Thus, both subtypes were probably derived from a common ancestor. In other fish species, additional subtypes of *Teratorn*-like viruses were identified (Fig. 3*C*) (Inoue et al. 2018).

In addition to transposase, about 90 other genes were predicted in *Teratorn* elements (Fig. 3*C*). Some of these are intact herpesvirus genes encoding functions required for virus propagation, such as DNA replication (DNA polymerase, primase, and UL21 homolog DNA helicase), virion maturation (capsid maturation PR), viral DNA packaging (large subunit terminase), and structural proteins (major capsid protein, subunit 2 capsid triplex protein, and Env glycoprotein). Others bear sequence similarity to genes from other organisms, which may have been secondarily obtained from infected host genomes or from other sources, for example, bacteria and viruses. Thus, these elements may also be regarded as potential HGT vehicles.

*Teratorn*-like viruses are widely distributed in teleosts (Aswad and Katzourakis 2017; Inoue et al. 2018). At least 15 out of 54 examined teleost genomes harbor viral-like DNA-polymerase-containing sequences (Aswad and Katzourakis 2017). An exhaustive search shows that 22 out of 77 fish genomes contain *Teratorn*-like sequences, some of them in multiple copies (Inoue et al. 2018). Interestingly, *Teratorn*-like viruses were found only in teleosts but not in Chondrichthyes, Sarcopterygii or amphibians. Overall, the long-term coexistence and coevolution of *piggyBac*-like elements and *Teratorn*-like viruses represent examples of successful fusion of two genetic entities which allowed herpesviruses to become endogenous viral elements integrated into teleost genomes.

## Could Large Eukaryotic TEs Facilitate Gene Transfer?

Although composite mobile units of 200–300 kb, often framed by HERV sequences, have been reported in the human genome (Ji and Zhao 2008), subsequent studies classified them as segmental duplications (Mohajeri et al. 2016); thus, we are not considering them here in detail. In the mid-1980s, similarly long units of mobility spanning hundreds of kilobases were described for the composite Ising TE (Transposing Element) of *Drosophila melanogaster* mobilizing cytologically visible DNA segments located between foldback DNA transposons (Ising and Block 1984; Lovering et al. 1991). Duplication of large genic regions and their relocation to different chromosomal positions can enable subfunctionalization and neofunctionalization of host genes (Lynch and Conery 2000)—a reliable but slow and difficult route for bringing innovation into the genome.

As an evolutionary shortcut, the HGT route for acquisition of the preexisting genetic material from elsewhere may look easier than lineage-specific duplications followed by gradual diversification. Moreover, if the function to be acquired is multicomponent, for example, a biosynthetic cluster, it is hard to envision its gradual evolution, as opposed to acquisition of the entire block. Examples of such acquisition include metabolic gene clusters in fungi, whereby large chromosomal segments encoding a physically linked set of preassembled components of biosynthetic pathways can be transferred via HGT (Wisecaver and Rokas 2015). In bacteria, delivery of a 58-kb secondary metabolism cluster into heterologous hosts could be achieved by transposition (Fu et al. 2008). However, transfer of such segments in eukaryotes requires overcoming of numerous barriers nonexistent in bacteria (nuclear membranes and chromatin, promoter and splicing signal compatibility, metazoan germ-line protection, etc.).

It is hardly a coincidence that in eukaryotes metabolic gene clusters are predominantly found in subtelomeres (Keller et al. 2005), which are characteristically rich in TEs. Notably, terminal location offers an easy way to diploidizing a newly acquired stretch of DNA via break-induced replication, whereas internal long stretches of internal nonhomology can easily cause chromosome pairing issues. TEs are often found in association with horizontally transferred genes in bdelloid rotifers, as well as with secondary metabolism clusters and pathogenicity determinants in fungi, where they promote segmental duplications and inversions (Flot et al. 2013; Grandaubert et al. 2014; Dallery et al. 2017). However, direct participation of TEs in cluster relocation remains to be demonstrated.

Overall, the potential of large TEs, especially virus-like ones, to facilitate HGT should not be overlooked. Horizontal transposon transfer is widespread and applicable to class I and class II TEs, and either class may utilize viruses as vectors (Gilbert and Cordaux 2017; Gilbert and Feschotte 2018). Furthermore, the essentially viral nature of class I LTR retrotransposons (often harboring *env* genes, which equalize them with endogenous retroviruses) or class II Polintons (encoding major and minor icosahedral virus capsid proteins) may endow them with autonomous ability to cross species boundaries (Malik et al. 2000; Krupovic et al. 2014; Rodriguez et al. 2017). Additionally, ssDNA viruses, despite their small genomes, might provide another avenue for gene transfer, relying on Rep proteins for rolling-circle replication and integration (Liu et al. 2011; Krupovic and Forterre 2015). Equally dependent on Rep function are *Helitrons*, which can also be transmitted by large DNA viruses (Thomas et al. 2010; Coates 2015; Heringer et al. 2017). It is noteworthy that many additional domains found in large TE ORFs, as well as accessory TE ORFs, are often shared between TEs and eukaryotic viruses, forming an amalgam of accessory functions that may be commonly used by both, and indicate frequent exchanges.

If transmission relies on nucleic acid encapsidation, which could also provide a certain level of protection from environmental degradation, then the size of transferred segments may be limited by the packaging capacity of the corresponding virus-like entity, which for RNA viruses should be lower than for DNA viruses. As mentioned above, such limits for RNA-based elements now appear to be in the range of 30–40 kb, whereas DNA-based ones may shuttle up to a few hundred kilobases. However, unless the HGT event is very recent, any molecular signatures of short *cis*-acting sequences that may have facilitated transfer of gene blocks could be rapidly erased or become unrecognizable if the initially responsible *trans*-competent TE is lost from the genome (Arkhipova et al. 2013). Moreover, ongoing TE activity tends to disrupt synteny in gene clusters. Thus, for a TE-mediated HGT event to be caught in the act, several conditions should be met. The full-length autonomous TE or its close relatives should still be present to help delineate the TE ends and involvement. These TE features might however be quickly lost by mutation, and furthermore loss of mobility is usually a prerequisite for domestication, providing protection from RNA-mediated silencing mechanisms. Any horizontally transferred gene might also quickly suffer pseudogenization and not be identifiable unless it is beneficial and purifying selection acts on the gene. So, there may be only a short window of time for bona fide HGT mediated by TEs to be identified.

## Concluding Remarks

Evolution of the simplistic views on eukaryotic TE organization gradually led to realization of their largely modular structure, whereby INs of different types may be combined with different replicases and diverse accessory functions to achieve mobilization of DNA segments of increasing size ranges. Successful domain combinations may emerge at any point in evolution: Early emergence leads to more widespread taxonomic distribution, whereas taxon-specific combinations may be able to spread either vertically or horizontally. Such molecular combinatorics involves functional modules with different evolutionary histories and globally results in a network-like pattern of inter-TE relationships, which parallels and, in many cases, converges with a similar pattern observed in the viral world (Koonin et al. 2015; Iranzo et al. 2016). The boundary between viruses and TEs is highly flexible, and the balance is easily shifted toward viruses through encapsidation and intercellular transmission functions, or toward TEs through integration/excision functions. Third-generation sequencing of understudied taxa should uncover even more diverse TEs, some of which could display such dual nature, offering better opportunities to serve as transfer vectors. Although the emergence of distinctive eukaryotic cellular features imposed new demands on transfer functions, the same modular principles also operate in prokaryotic MGEs, where integration can be combined with conjugation, and transfer modules can be incorporated from plasmids. Accessory functions may be acquired on a case-by-case basis from the hosts or from other mobilome components and may help to optimize the core “selfish” functions such as integration and transfer, or aid in regulation and in host–TE interaction. Sometimes, it may be difficult to distinguish between accessory and cargo proteins, as the functional roles of the captured genes may often be conditional, and their source is not always evident. However, acquisition of novel functions enabling expansion of mobilizable units may facilitate not only TE survival but also interspecific genetic exchange, and would ultimately serve an important role in evolution and adaptation by generating novelty and diversity. 
